# Personalized Dendritic Cell Vaccines—Recent Breakthroughs and Encouraging Clinical Results

**DOI:** 10.3389/fimmu.2019.00766

**Published:** 2019-04-11

**Authors:** Beatris Mastelic-Gavillet, Klara Balint, Caroline Boudousquie, Philippe O. Gannon, Lana E. Kandalaft

**Affiliations:** Department of Oncology, Center for Experimental Therapeutics, Ludwig Center for Cancer Research, University of Lausanne, Lausanne, Switzerland

**Keywords:** dendritic cells, vaccines, cancer, immunotherapy, neo-antigens

## Abstract

With the advent of combined immunotherapies, personalized dendritic cell (DC)-based vaccination could integrate the current standard of care for the treatment of a large variety of tumors. Due to their proficiency at antigen presentation, DC are key coordinators of the innate and adaptive immune system, and have critical roles in the induction of antitumor immunity. However, despite proven immunogenicity and favorable safety profiles, DC-based immunotherapies have not succeeded at inducing significant objective clinical responses. Emerging data suggest that the combination of DC-based vaccination with other cancer therapies may fully unleash the potential of DC-based cancer vaccines and improve patient survival. In this review, we discuss the recent efforts to develop innovative personalized DC-based vaccines and their use in combined therapies, with a particular focus on ovarian cancer and the promising results of mutanome-based personalized immunotherapies.

## Introduction

Dendritic cells (DC) are the most potent professional antigen-presenting cells (APC) and play critical roles in regulating the innate and adaptive immune responses (1). In their immature state, DC patrol the tissue microenvironment and become activated in the presence of foreign pathogens. This activation occurs following stimulation by exogenous danger signals via pattern recognition receptors (PRR) such as Toll-like receptors (TLR) (2, 3) and leads to DC migration to the draining lymph node and the presentation of the processed epitopes to T cells (4). During the T cell activation, DC engage the T-cell receptor (TCR), secrete specific cytokines and stimulate the immune responses toward TH1, TH2, or Tregs depending on the cytokine environment. Due to their proficiency at antigen cross-presentation (i.e., the presentation to both CD4^+^ and CD8^+^ T cells), DC have been used as vaccine platforms to induce anti-tumor cytotoxic T lymphocyte (CTL) CD8 immune responses (5–8).

Various types of DC-based vaccines have been evaluated in clinical trials. The most commonly used preparation involves the reinfusion of *ex-vivo* derived DC pulsed with tumor-associated antigens (TAAs) or tumor cell lysates and stimulated with a defined maturation cocktail. In the earlier trials, the gold standard maturation cocktail included the pro-inflammatory cytokines TNF-α, IL-1β, and IL-6 in combination with prostaglandin E2 (PGE2) (8–10). However, despite the important roles of PGE2 in promoting DC migration (11) and in enhancing T cell proliferation (12), it has also been shown that PGE2 may induce differentiation of regulatory T cells (13), increase the expression of the pro-tolerogenic enzyme indoleamine 2,3-dioxygenase (IDO) (14), and may limit IL-12p70 production (15). As these PGE2-related activity may curtail the anti-tumoral immune response, alternative methods of *ex vivo* maturation of DC have been explored such as the triggering of co-stimulatory pathways (e.g., CD40-CD40L) (16) and the activation of the TLR using agonists such as poly IC (TLR3) (17), resiquimod (TLR7/8) (8) and 3-O-deacylated monophosphoryl lipid A (MPLA) (18), a modified TLR4 agonist with less toxicity than LPS. Moreover, DC subsets have been directly targeted *in vivo* by administration of TAAs directly to DC or by intra-tumoral administration of immunomodulatory molecules to activate local DC.

Although, DC-based vaccinations looked promising after Sipuleucel-T (Provenge®) approval in 2010, a DC-based immunotherapy for the treatment of advanced prostate cancer (19), unfortunately, the vaccination against established malignancies has generally shown limited clinical benefit. There are a number of potential factors that can impact the efficiency of DC-based vaccines. For instance, there is a reduction TAAs expression by tumor cells leading to immunosuppression and the immune evasion of cancer cells. Tumor cell elimination may also be blunted by the immune suppressive barriers overexpression, such as checkpoint receptor signaling (CTLA-4, PD-1/PD-L1) and immunomodulatory cellular subsets [Tregs and myeloid-derived suppressor cells (MDSCs)] (20, 21). Moreover, there are evidences of defects in both the number and functions of DC subsets, which facilitate tumor progression and immune evasion (22–29). Overall, the transition of DC from an *in vitro* cell culture to an *in vivo* immunosuppressive environment may alter the effectiveness of DC-based immunotherapy.

Therefore, ongoing trials using DC-based vaccines are evaluating the use of combined immunotherapies to favor DC activation and promote T cell functions, and overcome tumor immune evasion. The Indian government agency (CDSCO-Central Drugs Standard Control Organization) recently approved in 2017 an autologous monocyte-derived and tumor lysate-pulsed mature DC-based vaccine (APCEDEN®) for treatment of four cancer indications (prostate, ovarian, colo-rectal and non-small cell lung carcinoma) (30). The multicentric phase II clinical trial by Bapsy et al. (31) demonstrated that this formulation was safe and well-tolerated in patients with refractory solid tumors. Moreover, the efficacy profile of APCEDEN® therapy demonstrated a survival benefit of >100 days (30).

## Human Blood Dendritic Cells

DC originate from the common myeloid bone marrow progenitor cells and can be found in both, lymphoid and non-lymphoid tissues in an immature state (1). DC are heterogeneous and consist of multiple specialized subtypes, which are defined based on their phenotypic and functional characteristics, including morphology and immunological features (expression of surface markers, cytokines, chemokines, and transcription factors). The homology of human DC and mouse DC populations have been extensively studied using transcriptional profiling (32–36). In humans, all DC express high levels of MHC class II molecules (HLA-DR), and lack lineage-specific surface markers for T cells (CD3), B cells (CD19/20), and natural killer cells (CD56). The DC subtypes found in the blood are myeloid DC (mDC) (also termed CD11c^+^ conventional DC, cDC), which can be further divided into CD141^+^ mDC, CD1c^+^ mDC, and CD123^+^ plasmacytoid DC (pDC) (37). The CD1c^+^ mDC account for the majority of the mDC population in the human blood representing approximately 1% of all mononuclear cells, with the CD141^+^ mDC representing only 0.1%. Compared with CD141^+^ mDC, the CD1c^+^ mDC have an inferior capacity to cross-present antigen to CD8^+^ T cells (35, 38). Human CD141^+^ DC are homologous to the mouse cross-presenting CD8α^+^/CD103^+^ DC, and are characterized by the exclusive expression of XCR1 and Clec9A (33, 39–43). The pDC are specialized producers of type I interferons in response to viruses (44) and can, on one end, induce Tregs expansion and tolerance (45, 46), while effectively cross-present antigens to CTL (47–49). Using mass cytometry (i.e., CyTOF), Guilliams et al. identified that the combination of the two markers (CADM1 and CD172a) could be used as flow cytometry markers to identify the conventional subsets of mDC across tissues and species (human, macaque and mouse) (50). Thus, CD141^+^ DC can be defined as CADM1^hi^CD172a^lo^, while the CD1c^+^ mDC correspond to CADM1^lo^CD172a^hi^ cells. Notably, the conventional identification of mDC or pDC (37) has lately been challenged by a study, which, using single-cell transcriptome profiling, demonstrated that human blood DC could be further stratified into six distinct populations (51). This increasing knowledge about DC subsets will certainly be exploited for the design of novel strategies to improve the clinical efficacy of cancer vaccines.

The isolation of DC subset is another for the generation of DC-based vaccine has also improved over the years. Initially, DC subsets were isolated directly *ex vivo* from the peripheral blood to produce DC-based vaccines for immunization of B cell-lymphoma patients against their TAAs (52). As DC have a low frequency in peripheral blood, low numbers of DC were isolated using this method. Nowadays, most clinical studies employ monocyte-derived DC (MoDC) in the generation of DC-based vaccine because of the relative ease at obtaining sufficient number of cells from peripheral blood and their functionality (53, 54). MoDC are a subset of DC exhibiting common features with cDC (55), including the ability to migrate, to potently stimulate CD4^+^ and CD8^+^ T cells, to produce key cytokines (IL-1, IL-6, TNF-α, IL-12, and IL-23) (56), and to express cell surface markers such as CD11c and MHC II (55). Autologous MoDC can be obtained by culturing human peripheral blood monocytes (CD14^+^) in the presence of GM-CSF and IL-4 (57) with the resulting vaccines eliciting tumor-specific T cell responses and some clinical efficacy (56).

With recent technological advances in isolation of specific immune cell populations, second generation DC vaccine have focused on the collection of blood-derived primary DC subsets. As previously mentioned, naturally circulating DC have a low frequency in peripheral blood (<1% of leukocytes). Nonetheless, there exist significant transcriptional and functional differences between the blood-derived DC in comparison with the *in vitro* generated MoDC suggesting that blood-derived DC may be superior for therapeutic vaccination (32, 58). Early phase I results suggest that vaccination with peripheral blood-derived pDC or mDC is safe and well-tolerated amongst patients with advanced-stage melanoma (59), prostate carcinoma (60) or acute myeloid leukemia (61). One such trial is based on a novel type of blood-derived DC vaccine is being assessed within the collaborative European project entitled “Professional cross-priming for ovarian and prostate cancer” (PROCROP). For this trial, a CD141^+^ subset of blood-derived mDC, which has superior capacities at cross-presenting TAAs to CD8^+^ T cells (39, 42, 62), is being evaluated as a personalized DC vaccine.

Altogether, clinical trials have yet to prove that blood-derived DC vaccines are more efficacious than *in vitro* generated MoDC (63). For instance, the development of second generations of DC-based vaccines may also face multiple technical challenges such as the limited availability of cells that can be purified, the large amount of blood or leukapheresis to be collected, and the negative effects of chemotherapy that may reduce the number of DC in the peripheral blood (64).

## Dendritic Cell Dysfunction in Cancer

Optimal DC function is necessary for the initiation of protective anti-tumor immunity. Yet, it is known that immunosuppressive factors expressed by the tumors cells, including IDO (65, 66), Arginase I (67), IL-10 (68, 69), TGF-β (23, 70), PGE2 (71, 72), and VEGF (73–77), can impair the differentiation, maturation, and function of the host DC (78–80), which may become tolerogenic and favor the stimulation of regulatory T cells (81, 82). For instance, high level of intratumoral pDC is associated with poor disease outcome across several tumor types (83, 84). The impairment of DC differentiation (80, 85), and the resulting inadequate antigen-presenting functionality of DC, contributes to T cell anergy or exhaustion is well documented in cancer. In a breast and pancreatic cancer study, tumor-derived granulocyte-stimulating factor induced alterations in the development of CD141^+^ DC, which were associated with impaired CD8^+^ T cell responses and correlated with poor clinical outcomes (86). An additional mechanism contributing to the impaired antigen processing ability of intra-tumoral DC is the accumulation of pathological amount of lipid by the DC due to up-regulated expression of scavenger receptor A (SR-A) (87). These lipid-laden DC have reduced capacity to stimulate allogeneic T cells (87).

It was previously demonstrated that DC derived from patients with advanced cancer are weak stimulators of T cells compared to healthy volunteers (88). In some tumors, as cancer progresses, tumor-infiltrating DC accumulate and switch from immunostimulatory to regulatory phenotypes (23), and correlates with the increased expression of negative costimulatory molecules such as TIM3 (89), PD-L1 and PD-1 (90) as well as the production of L-Arginase (91). In fact, this is a predominant mechanism of DC dysfunction in ovarian carcinoma, with PD-1^+^ PD-L1^+^ CD277^+^ DC accumulating in the tumor over the course of the disease (90, 92). The increased expression of PD-1 was shown to affect the function of DC by inhibiting NF-κB activation, and was associated with decreased T cell activity and reduced tumor-infiltrating T cells in advanced cancer (93). CD277 was shown to be universally expressed in ovarian cancer-infiltrating DC and may affect the expansion of TCR-stimulated T cells.

Therefore, the immunosuppressive DC, controlled by the tumor microenvironment, plays an important role in supporting tumor progression, and probably limiting the success of DC-based vaccine in cancer patients. There is increased awareness on the influence of age-related changes on the development of tumors and on treatment prognosis. Aging has already a profound effect on DC function, affecting numbers and functions of pDC (94), and inducing substantial changes in gene expression profile of CD1c^+^ DC as illustrated by significant down-regulation of antigen presenting and energy generating genes (95). Thus, to overcome systemic immune dysfunction and augment DC-induced responses *in vivo*, many investigators are combining DC-based vaccines with tumor-damaging agents or considering the use of DC-based vaccines to treat earlier in the course of the disease (96). Notably, combining CD40 agonists with TLR3 activation was shown to be sufficient to reverse the immunosuppressive phenotype of tumor-infiltrating DC into APCs capable of priming anti-tumor T cell responses (97).

## Active Ingredients of DC-based Cancer Vaccines

### Tumor Antigens

TAAs are a crucial component of DC vaccines as they represent the targets for CTL-generated anti-tumor immune response. Non-mutated self-antigens resulting from over-expression of tissue- or lineage-specific genes induced by transformation induce low T cell reactivity due to central tolerance mechanisms. Conversely, mutated neo-antigens are generated by somatic mutations due to the tumors' inherent genetic instability rendering them tumor-specific and private, with the advantage of being recognizable for T cells and not impacted by central tolerance.

### Defined Antigens

The most widely used cancer vaccines tested so far were based on defined, shared TAAs (e.g., MART-1, gp100, CEA, PSA, p53, NY-ESO-1, MAGE-A3), which are HLA restricted (98–103). Both, individual and the combination of several defined antigens were tested, but only achieved limited clinical efficacy (104–106). A potential disadvantage of immunotherapy targeting one or few defined TAAs is the possibility of rapid development of tumor escape variants that lose the expression of these epitopes (107). Using multiple (defined or undefined) antigens as vaccine targets may be crucial for achieving significant clinical benefit and may overcome the challenge of tumor escape via antigen-loss.

### Neo-Antigen-Targeted Approaches

The high mutational rate of tumor cells results in the expression of neo-antigens that are tumor specific. The identification of patient specific TAAs, including both shared tumor antigens and neo-antigens, is now possible using next-generation sequencing (NGS) and bioinformatics tools (e.g., NetMHC) (108) complemented or not by direct isolation of HLA-bound peptides (immunopeptidome) and mass spectrometry (MS) analysis (109). The personalized cancer vaccine can be manufactured based on neo-antigens that have been identified and used to manufacture peptides or RNA for the pulsing of DC. Nonetheless, two major challenges arise from this approach: the time between tumor resection and first vaccine injection, which can reach several months, and the cost of the neo-antigen identification process.

Three recent Phase I clinical trials confirmed promising potential of personalized cancer vaccines based on neo-antigens (110–112), with the study by Carreno et al. utilizing DC-based vaccine (110). Whole-exome sequencing was carried out to identify somatic mutations in tumors from three patients with melanoma and short peptides coding for seven neo-antigens were pulsed onto autologous DC. Despite the small sample size, the study proved that neo-antigen cancer vaccines could elicit neo-antigen specific T cell response with some patients showing stabilized or non-recurrent disease (110).

### Whole Tumor Preparations

In indications where surgery can be performed as part of the treatment, the resected tumor tissue can be used as a source of patient-specific TAA by preparing a tumor cell lysate. Alfaro et al. used freeze-thaw lysis from biopsies to generate glioma-specific lysate (113). The treatment induced IL-12 production in each patient and circulating tumor cells markedly dropped in 6 of 19 cases with five patients experiencing disease stabilization (114). The immunogenicity of tumor cell lysate can be enhanced using alternative lysate preparation methods such as freeze-thaw, UV irradiation or oxidation treatment (115–120). Our group showed that tumor cells oxidation using hypochlorous acid (HOCl) combined with freeze-thaw cycles results in primary necrosis of tumor cells, and increases immunogenicity of the resulting tumor lysate (121). The main advantages of using autologous tumor lysate as a source of TAAs are the absence of HLA restriction and the reduced time and cost of manufacturing in comparison to the neo-antigen prediction strategies.

## Recent Accomplishments in Personalized DC-based Immunotherapy

### Current Treatment Strategies for Advanced Ovarian Cancer

A DC-based vaccine generated by differentiation of autologous Mo-DC pulsed with HOCl oxidized autologous tumor cell lysate (OC-DC vaccine) was tested in platinum-treated, immunotherapy-naïve, recurrent ovarian cancer patients in a single-center, multi-cohort, non-randomized phase I trial (122). During the study, a total of 392 vaccine doses were administered intra-nodally under ultrasound guidance without serious adverse events. The results of the first of three cohorts was reported by Tanyi et al. (122). In this study, the DC-based vaccine was administered either alone, in combination with bevacizumab or in combination with bevacizumab and low-dose intravenous cyclophosphamide until disease progression or vaccine exhaustion. This OC-DC vaccine induced T cell responses (increased in IFN-γ production) to autologous tumor antigens, which were detected in 11 of 22 evaluable patients on week 12. Moreover, this antitumor immune response was associated with significantly prolonged survival with increased neo-antigen specific T cells responses, both previously recognized and non-recognized neo-epitopes.

Overall from the 25 patients treated two (2) patients showed partial response and 13 patients experienced stable disease, which persisted for a median of 14 months from enrolment. Of note, vaccine responders experienced significantly longer progression-free survival (PFS) compared to non-responders patients. The 2-year overall survival (OS) rates of the responder patients was 100%, whereas the 2-year OS of non-responders was 25%. The best results were obtained with the triple combination of vaccine plus bevacizumab and cyclophosphamide. This study demonstrated that the use of OC-DC vaccine was safe and elicited a marked antitumor immunity, including tumor-specific neo-antigens. Altogether, personalized DC vaccines using whole tumor lysate can drive responses to private antigens and, in combination with other immunotherapy treatments, can greatly improve clinical outcome.

### Promising Phase 3 Studies in Progress

An exhaustive list of DC-based studies is available in Table 1. Notably, a phase 3 trial is currently testing DC vaccine loaded with autologous tumor lysate (DCVax-L) in patients with newly diagnosed glioblastoma following surgery as add-on to the standard of care combining radiation and chemotherapy (NCT00045968; Northwest Therapeutics). Patients are receiving temozolomide plus DCVax-L (*n* = 232) or temozolomide and placebo (*n* = 99). DCVax-L is administered intra-dermally six (6) times the first year and twice per year thereafter. Following recurrence, all patients are allowed to receive DCVax-L. The first reported results showed that the median OS was 23.1 months from surgery as compared with the 15-17 months achieved with SOC only in past studies (123). Only 2.1% of patients had a grade 3 or 4 adverse event related to the vaccination treatment. Due to its safety profile, this DC vaccine has the potential to be administered in a wide range of indications and applied in a wide range of combinations.

**Table 1:** Table of current ongoing clinical trials using personalized DC-based vaccines.

		**NCT number**	**Indication**	**Interventions**	**Phase**	**Enrollment**	**Start date**	**Estimated primary completion date**
Tumor lysate	1	NCT00703105	Ovarian cancer	Ontak (anti-CD25)|DC vaccine + ontak	Phase 2	36	2008	2018
	2	NCT01204684	Glioma|Astrocytoma|Astro-oligodendroglioma|Glioblastoma	Autologous tumor lysate-pulsed DC + 0.2% resiquimod|DC vaccination + polyICLC	Phase 2	60	2010	2018
	3	NCT01635283	Newly diagnosed or recurrent low-grade glioma	Tumor lysate-pulsed autologous DC vaccine	Phase 2	18	2012	2019
	4	NCT01946373	Malignant melanoma	Cyclophosphamide|Fludarabine|T cells|Interleukin-2|DC vaccine	Phase 1	10	2013	2018
	5	NCT01973322	Malignant melanoma stage III|Stage IV	Arm 1: autologous DC loaded with autologous tu lysate (DC vaccine) + RT|Arm 2: DC vaccine + IFN-α|Arm 3: both arm 1 and 2 + RT|Arm 4: DC vaccine	Phase 2	24	2013	2019
	6	NCT01957956	Newly diagnosed glioblastoma	Tumor lysate-pulsed autologous dendritic cell vaccine + temozolomide	Early phase 1	21	2013	2016
	7	NCT01808820	Malignant glioma|Glioblastoma	Dendritic cell vaccine|Tumor lysate|Imiquimod|Leukapheresis	Phase 1	20	2013	2019
	8	NCT02496520	Advanced solid tumors, sarcoma|Central nervous system tumor	Dendritic cells|Surgery as needed|Chemotherapy as needed|Radiation: radiation therapy as needed	Phase 1|2	10	2014	2018
	9	NCT01803152	Sarcoma|Soft tissue sarcoma|Bone sarcoma	Biological: dendritic cells vaccine|Lysate of tumor|Gemcitabine|Imiquimod|Leukapheresis	Phase 1	56	2014	2019
	10	NCT02718391	Malignant melanoma	DC pulsed with autologous tumor lysate	Phase 2	120	2015	2019
	11	NCT02301611	Malignant melanoma	Autologous Tumor Lysate (TL) + Yeast Cell Wall Particles (YCWP) + Dendritic Cells (DC) (TLPLDC Vaccine)|Placebo	Phase 2	120	2015	2019
	12	NCT02503150	Metastatic colorectal cancer	Antigen pulsed dendritic cells + chemotherapy|Chemotherapy	Phase 3	480	2015	2019
	13	NCT02678741	Metastatic melanoma	TLPLDC vaccine in addition to standard of care checkpoint inhibitor of choice	Phase 1|Phase 2	45	2016	2019
	14	NCT03395587	Newly diagnosed glioblastoma	Autologous DC pulsed with autologous tumor lysate	Phase 2	136	2018	2022
	15	NCT03360708	Recurrent glioblastoma	Cytokine-induced killer cells|Tumor lysate-pulsed autologous DC vaccine	Early phase 1	20	2018	2022
	16	NCT03014804	Recurrent glioblastoma	Autologous dendritic cells pulsed with tumor lysate|Nivolumab	Phase 2	30	2018	2020
RNA	17	NCT01983748	Uveal melanoma	Autologous DC loaded with autologous tumor RNA	Phase 3	200	2014	2022
Peptide	18	NCT02775292	Adult solid neoplasm|Childhood solid neoplasm|Metastatic neoplasm	Aldesleukin|Cyclophosphamide|Fludarabine phosphate|Nivolumab|NY-ESO-1 reactive TCR retroviral vector transduced autologous PBL|NY-ESO-1(157-165) peptide-pulsed autologous DC vaccine	Phase 1	12	2017	2019
Tumor neoantigen	19	NCT01885702	Colorectal cancer	Neoantigen-loaded DC vaccination	Phase 1|2	25	2010	2016
	20	NCT03300843	Melanoma|Gastrointestinal|Breast| Ovarian|Pancreatic cancer	DC vaccine loaded with neoantigen coding peptide	Phase 2	86	2018	2027

Another phase 3 study is currently evaluating the efficacy adjuvant vaccination using RNA-loaded autologous DC vaccine to treat patients with uveal melanoma (NCT01983748). This study will compare standard of care treatment with vaccination (8 intravenous of vaccine over 2 years).

Finally, a phase 3 study is currently evaluating active immunization in adjuvant therapy of patients with stage 3 melanoma with natural (BDCA3^+^) dendritic cells (nDC) pulsed with peptides (NCT02993315). Patients will receive nDC vaccine by three (3) intranodal injection per cycle for a maximum of three (3) cycles or placebo injections to determine if adjuvant nDC vaccination improves 2-year RFS rate.

## Predictive Markers for the Clinical Efficacy of DC-based Vaccines

Another path to the improvement of DC-based vaccine efficiency is based on the identification of surrogate biomarkers of the triggered immune response against the tumor that would strongly and uniformly correlate to vaccine efficacy. Studies have identified different potential biomarkers of clinical responses to DC-based vaccination. For instance, in melanoma, two (2) candidate genes were identified with a predictive value for a positive outcome to a DC-based immunotherapy (124). The chemokine receptor CXCR4 and the receptor for the FC portion of IgD (CD32) were over-expressed in the lymphocytes cell membranes and in the monocyte populations in immunological responder patients as compared to non-responder patients (124). Higher CXCR4 protein expression was found in CD8^+^ T cells pre- and post- whereas higher CD32 protein expression in monocyte populations was identified in responder patients at pre-treatment time points (124). In a recent phase II study in patients with glioblastoma, DC vaccination induced a significant and persistent activation of CD56^dim^ cytotoxic NK cells, whose increased response was strongly associated with prolonged survival, while CD8^+^ T cells had only a poor contribution to anti-tumor responses (125). In NSCLC patients, the survival time was closely associated with the BDCA1^+^ DC/BDCA3^+^ DC ratio in peripheral blood after DC immunotherapy (126).

Tumor-infiltrating lymphocytes (TIL) are examined extensively in various cancer types, including epithelial ovarian cancer, with their presence found to be an important prognostic factor (127–134). Additionally, in ovarian cancer, infiltrating Tregs in the tumor microenvironment correlate with poor prognosis (135–137). In the context of DC-vaccination, in glioma, the TIL content was identified as a predictor of clinical response (138). An increased overlay in the TCR repertoire of TIL and circulating T cells correlated with improved responses to DC-based vaccination and overall survival (138). Hence, the TIL content may be used as a selection tool to identify patients who could potentially benefit from DC vaccination therapy.

In terms of monitoring anti-tumor vaccine trials, a study by Kirkwood et al. found that functional assessment of T cells such as interferon-γ production is preferable as opposed to frequency or phenotype of effector T-cells (139). In a multicenter study (ECOG E1696), where melanoma patients were treated with a peptide vaccine, there was a significant difference in OS by immune response status. Immune responders, patients whose T cells exhibited interferon-γ response (against to one or more of the three antigens measured by ELISPOT) lived longer than the nonimmune responders (median OS, 21.3 vs. 10.8 months; *P* = 0.033).

In conclusion, highly reliable molecular or cellular biomarkers of the clinical efficacy of personalized DC-based vaccines are still missing. Prospective longitudinal studies will help identify predictive prognostic and treatment-efficacy biomarkers using “Omics” data (140) and systems biology analysis. Therefore, there is an urgent need for clinical studies beyond phase II to demonstrate that DC-based vaccines can induce durable objective responses and improve long-term survival in cancer patients, and maybe identify strong correlate for all malignancies.

## Conclusions

The development and success of DC-based immunotherapies has been hampered by several factors; (1) the immunosuppressive tumor microenvironment, particularly in advanced stage of the disease (2) the limited capacity of systemically administered DC to localize to the tumor-draining lymph nodes, (3) the low avidity of TAAs-specific T cells, and (4) the lack of reliable prognosis biomarkers. The rapidly increasing knowledge about DC subsets and the tumor-induced suppressive microenvironment must be exploited to design novel and improved cancer vaccines. The future of DC vaccines will certainly rely on combination therapies. As discussed in this review, recent studies have shown the great potential of such strategies, especially when using personalized DC vaccines. Overcoming the cancer immunosuppressive environment will reveal the real therapeutic potential of such DC vaccine.

## Author Contributions

BM-G and KB wrote the manuscript. All authors, BM-G, KB, CB, POG, and LEK contributed to manuscript revision, read and approved the submitted version.

### Conflict of Interest Statement

The authors declare that the research was conducted in the absence of any commercial or financial relationships that could be construed as a potential conflict of interest.

## Abbreviations

DC: Dendritic cell
APCs: Antigen-presenting cells
CTL: Cytotoxic T lymphocyte
TAAs: tumor-associated antigens
MoDC: Monocyte-derived DC
OS: Overall survival
TILS: tumor infiltrating lymphocytes.
